# Cardiac Autonomic Modulation Is Determined by Gender and Is Independent of Aerobic Physical Capacity in Healthy Subjects

**DOI:** 10.1371/journal.pone.0077092

**Published:** 2013-10-03

**Authors:** Sabrina G. V. Dutra, Ana Paula M. Pereira, Geisa C. S. V. Tezini, José H. Mazon, Marli C. Martins-Pinge, Hugo C. D. Souza

**Affiliations:** 1 Department of Biomechanics, Medicine and Rehabilitation, School of Medicine of Ribeirão Preto, University of São Paulo, Ribeirão Preto, São Paulo, Brazil; 2 Department of Physiological Sciences, State University of Londrina, Londrina, Paraná, Brazil; Northwestern University, United States of America

## Abstract

**Background:**

Aerobic physical capacity plays an important role in reducing morbidity and mortality rates in subjects with cardiovascular diseases. This action is often related to an improvement in the autonomic modulation of heart rate variability (HRV). However, controversies remain regarding the effects of physical training on cardiac autonomic control in healthy subjects. Therefore, our objective was to investigate whether aerobic capacity interferes with the autonomic modulation of HRV and whether gender differences exist.

**Methods:**

Healthy men and women (N=96) were divided into groups according to aerobic capacity: low (VO_2_: 22-38 ml/kg^-1^ min^-1^), moderate (VO_2_: 38-48 ml/kg^-1^ min^-1^) and high (VO_2_ >48 ml/kg^-1^ min^-1^.) We evaluated the hemodynamic parameters and body composition. The autonomic modulation of HRV was investigated using spectral analysis. This procedure decomposes the heart rate oscillatory signal into frequency bands: low frequency (LF=0.04-0.15Hz) is mainly related to sympathetic modulation, and high frequency (HF=0.15-0.5Hz) corresponds to vagal modulation.

**Results:**

Aerobic capacity, regardless of gender, determined lower values of body fat percentage, blood pressure and heart rate. In turn, the spectral analysis of HRV showed that this parameter did not differ when aerobic capacity was considered. However, when the genders were compared, women had lower LF values and higher HF values than the respective groups of men.

**Conclusion:**

The results suggest that aerobic physical capacity does not interfere with HRV modulation; however, the cardiac modulatory balance differs between genders and is characterized by a greater influence of the autonomic vagal component in women and by the sympathetic component in men.

## Introduction

Low cardiorespiratory capacity is directly associated with an increase in the morbidity and mortality rates in both male and female subjects with cardiovascular diseases, regardless of the presence of other risk factors [1-3].

In this sense, the increase in aerobic capacity through the regular practice of physical exercises has been suggested to be a non-pharmacological means to prevent or treat a number of diseases, mainly those characterized as chronic degenerative, such as hypertension, diabetes mellitus and obesity [4,5].

In fact, there is evidence that moderate-to-high levels of cardiorespiratory capacity reduce the risks of mortality resulting from cardiovascular diseases or other causes in both genders, and this cardioprotective effect is independent of age, race, adiposity or other health conditions [3,6-9].

Despite this evidence, the causes of reduced morbidity via practicing physical exercises, which increases an individual’s aerobic capacity, have not been completely elucidated yet. It is common to report the improvement in cardiac autonomic control as one of the adaptations responsible for this reduction [10,11]. However, many clinical and experimental studies assessing autonomic control under different perspectives have shown conflicting results in terms of the adaptations induced by exercise on the autonomic modulation of heart rate variability (HRV) [12-15]. In fact, these conflicting results seem to be more related to healthy subjects because most studies demonstrate that the regular practice of physical exercises improves HRV in subjects with chronic degenerative diseases, such as hypertension, obesity and diabetes [10,11,13,16].

Moreover, some studies have reported that the higher level of aerobic capacity improves cardiac autonomic function in healthy subjects, suggesting the hypothesis that high-performance athletes have a better autonomic HRV modulation than sedentary subjects or those who practice physical activities for leisure and/or health reasons [17-19]. Another debate is whether the possible physical exercise-induced autonomic adaptations on HRV modulation would be the same for both genders [10,13,14,16,18] because very few studies have evaluated the differences in the cardiac autonomic modulation between healthy men and women with different levels of aerobic capacity. Our hypothesis is that sedentary men and women have different modulation patterns and that aerobic physical capacity can interfere with such patterns in a gender-specific way.

Therefore, the objective of the present study was to investigate whether different levels of aerobic capacity can influence cardiac autonomic modulation in healthy subjects and whether there are differences between men and women.

## Methods

### Participants

A total of 96 healthy subjects (53 men and 43 women), aged between 18 and 40 years, participated in the study. All subjects were normotensive and had normal cardiopulmonary function. The exclusion criteria were as follows: a high body mass (index > 25 kg/m^2^); the use of drugs or other addictive substances (tobacco and alcohol); a diagnosis of asthma, sleep apnea, or hypertension (blood pressure > 140/90 mmHg); metabolic disorders, including diabetes, dyslipidemia, pre-diabetes, and diabetes types 1 and 2; the chronic use of medications or substances that might interfere with cardiac autonomic function or the cardiovascular system; and the presence of musculoskeletal disorders that would prevent the subjects from performing the evaluations.

Prior to the experimental procedures, all volunteers were selected by anamnesis, which provided data regarding each participant’s physical fitness and health status. Regarding their athletic profile, the volunteers were questioned about their daily and weekly physical activities, including the type, frequency, and workload. All volunteers who were included in the “low aerobic capacity” group (sedentary) reported that they were not following any type of physical activity program and had only a small caloric expenditure in daily activities, such as walking and climbing stairs. All volunteers included in the “moderate aerobic capacity” (trained) group reported that they were following a physical activity program at the gym or outdoors once per day for 5 days per week, with an average time between 1 hour and 30 minutes and 2 hours. These volunteers were participating in amateur competitions, such as marathons, for recreational purposes and to maintain their health and quality of life. The volunteers included in the “high aerobic capacity” group (athletes) had daily training sessions (4-5 hours) divided into two periods (morning and night) 7 days per week to improve athletic performance. All volunteers in this group were elite athletes who had participated in national and international sports competitions in their respective modalities. The modalities practiced by both the “moderate” and the “high” groups were considered as *aerobic* in *nature* (long-distance races, such as marathons and triathlons). Finally, all the information regarding the athletic profile was compared with the results obtained from *cardiopulmonary stress testing* (ergospirometry).

After the screening, the selected volunteers were divided into three subgroups according to aerobic physical capacity, which was determined using a maximum ergospirometric test on a treadmill as follows: low aerobic capacity (VO_2max_: 22-38 ml kg^-1^ min^-1^), moderate aerobic capacity (VO_2max_: 38-48 ml kg^-1^ min^-1^), and high aerobic capacity (VO_2max_: > 48 ml kg^-1^ min^-1^).

Volunteers were screened at the Exercise Physiology Laboratory, Department of Biomechanics, Medicine and Rehabilitation, School of Medicine of Ribeirão Preto, University of São Paulo, Brazil. The study protocol was conducted in accordance with the ethical standards established by the Helsinki Declaration of 1975 and was approved by the Ethics Committee on Human Research, School of Medicine of Ribeirão Preto, University of São Paulo, Brazil (Protocol #4820/2011). All subjects were informed about the procedures and non-invasive experiments that would be performed in this study. After agreeing to participate in the study, all subjects signed an informed consent form.

### Study Design

Each volunteer was evaluated in the morning (08:00 to 10:00 am) during two laboratory visits. They were previously instructed not to perform intense physical activity and to avoid the consumption of alcoholic and caffeinated beverages for 48 hours prior to testing, and to sleep at least 8 hours and eat soft food 2 hours before testing.

### Hemodynamic parameters

Systolic arterial pressure (SAP), diastolic arterial pressure and mean arterial pressure (MAP) were obtained using a mercury column sphygmomanometer with the auscultatory method. Heart rate (HR) was obtained using an electrocardiographic digital recorder (ML866 PowerLab, ADInstruments, Bella Vista, Australia). Evaluations were performed before experimental procedures and after a 15-minute rest period.

### Spectral Analysis of HRV

HR recordings for HRV spectral analysis via electrocardiograms (ML866 PowerLab, ADInstruments, Bella Vista, Australia) were performed between 9:00 and 10:00 a.m. for 40 minutes while volunteers were in a supine position. Time series were obtained from adjacent R-R intervals (iRR) and then divided into segments of 200 beats, which were superimposed over the segments of 100 beats obtained from the previous series. After calculating the mean and variance of each segment, a spectral analysis was performed using the autoregressive model [20]. The oscillatory components present in the stationary segments from beat to beat of the iRR were calculated based on the resources of Levinson-Durbin, according to Akaike’s criteria [21]. This procedure allows the automatic quantification of the central frequency and influence of each relevant oscillatory component that is present in the interval series. The oscillatory components were classified as low frequency (LF) and high frequency (HF), and they showed fluctuations in the frequency ranges of 0.04-0.15 Hz and 0.15-0.5 Hz, respectively [20,21]. The power of the LF and HF components in the variability of the iRR was also expressed in normalized units and was obtained by calculating the percentage of variability in the LF and HF and considering the total power after subtracting the very low frequency (VLF) component (frequencies < 0.04 Hz). This normalization procedure minimizes the effects of total power changes on the variability of the absolute values of the LF and HF components [20,21]. Additionally, the LF/HF ratio was calculated to establish an index for the assessment of the cardiac autonomic modulation.

### Ergospirometric test

The maximum oxygen uptake (VO_2max_) was assessed by a maximal exercise test on a treadmill (Super ATL Millenium®, Inbramed/Inbrasport, Brazil) according to a previously published protocol [22]. The analysis of exhaled gases (O_2_ and CO_2_) was performed using a metabolic device (Ultima™ CardiO_2_, Medical Graphics Corp., St. Paul, MN, USA).

### Anthropometric parameters

Body mass index values were obtained using the formula W/H^2^, where W is the weight in kilograms and H is the height of the subject in meters. Body composition was evaluated using the bioelectrical impedance method (Quantum BIA 101; Q-RJL Systems, Clinton Township, MI, USA).

### Statistical Analysis

The results are shown as the mean ± S.E.M. (standard error of the mean). The effects of gender and aerobic physical capacity were assessed by two-way analysis of variance (ANOVA). When appropriate, post hoc comparisons were performed using the Student-Newman–Keuls test. Differences were considered significant when *p<0.05*. All statistical tests were performed with SigmaStat 3.5 software (Systat Software Inc., San Jose, CA, USA). The sample size calculation was performed using the “GraphPad StatMate 2.0”, confidence level of 95%, Power 80%, using the LF and HF variables in normalized units. It was been suggested the number of 12 volunteers in each group.

## Results

The characteristics and hemodynamic parameters of the subjects are presented in Table 1. The men had higher BMI values than women, regardless of the level of aerobic capacity (gender factor, F_(1,90)_: 27.9, p<0.001). In turn, the assessment of body fat percentage showed an inverse relationship with the level of aerobic capacity in both men and women. However, men had the lowest values at all levels of aerobic capacity. With regard to the hemodynamic parameters (Table 1), both men and women from the moderate and high aerobic capacity groups had lower HR values than the subjects from the low aerobic capacity group (aerobic physical capacity factor, F_(2,90)_: 71, p<0.001). However, there was no statistically significant difference between men and women when the groups were compared. An inverse relationship was observed between aerobic capacity and AP in both men and women (SAP, aerobic physical capacity factor, F_(2,90)_: 11.4, p<0.001; DAP, aerobic physical capacity factor, F_(2,90)_: 7.78, p<0.001; MAP, aerobic physical capacity factor, F_(2,90)_: 10.8, p<0.001). Additionally, women always demonstrated lower values than men (SAP, gender factor, F_(2,90)_: 18.9, p<0.001; DAP, gender factor, F_(2,90)_: 13.3, p<0.001; MAP, gender factor, F_(2,90)_: 18.2, p<0.001).

**Table 1:** Effects of gender and aerobic physical capacity on characteristics and hemodynamic values.

	**Low (N = 35)**					**Moderate (N = 36)**					**High (N = 25)**					**Gender Factor**			**Aerobic Physical Capacity Factor**			**Interaction**	
	**Men (n=18)**		**Women (n=17)**			**Men (n=22)**		**Women (n=14)**			**Men (n=13)**		**Women (n=12)**			**F_(DF)_**	***P***		**F_(DF)_**	***P***		**F_(DF)_**	***P***
**Characteristics**																							
Age, years	27 ±	1.2	27 ±	1.3		29 ±	1.4	27 ±	1.7		32 ±	2.1	31 ±	1.6		F_(1,90)_:0.28	*>0.05*		F_(2,90)_:4.68	*0.012*		F_(2,90)_:0.04	*>0.05*
Height, cm	177 ±	1.6	166 ±	1.3^a^		176 ±	1.4	165 ±	1.4^b^		175 ±	1.8	160 ±	2.5^c^		F_(1,90)_:84.9	*<0.001*		F_(2,90)_:2.78	*>0.05*		F_(2,90)_:0.71	*>0.05*
Weight, Kg	73 ±	1.7	61 ±	1.5^a^		74 ±	1.7	61 ±	1.4^b^		72 ±	2.1	54 ±	1.9 ^c e^		F_(1,90)_:101	*<0.001*		F_(2,90)_:3.9	*0.022*		F_(2,90)_:1.08	*>0.05*
BMI, Kg/m^2^	23.3 ±	0.3	21.9 ±	0.5^a^		23.7 ±	1.6	22.4 ±	0.4^b^		23.3 ±	0.5	20.8 ±	0.4^c^		F_(1,90)_:27.9	*<0.001*		F_(2,90)_:2.9	*>0.05*		F_(2,90)_:1.03	*>0.05*
% Body Fat	17.0 ±	0.3	21.7 ±	0.6^a^		13.0 ±	0.3^a^	13.9 ±	0.3^d^		10.1 ±	0.2 ^a b^	12.2 ±	0.4 ^c d e^		F_(1,90)_:70.8	*<0.001*		F_(2,90)_:258	*<0.001*		F_(2,90)_: 15.1	*<0.001*
VO_2peak_, ml Kg^-1^ min^-1^	31.7 ±	0.7	30.4 ±	0.6		43.6 ±	0.5^a^	41.8 ±	0.8^d^		62.1 ±	1.0 ^a b^	54.7 ±	1.5^c d e^		F_(1,90)_:0.08	*>0.05*		F_(2,90)_:268	*<0.001*		F_(2,90)_:2.66	*>0.05*
**Hemodynamic Values**																							
Heart Rate, bpm	81 ±	2.7	82 ±	2.8		65 ±	1.7^a^	68 ±	1.6^d^		52 ±	1.8 ^a b^	55 ±	2.0 ^d e^		F_(1,90)_:1.1	*>0.05*		F_(2,90)_:71	*<0.001*		F_(2,90)_:0.28	*>0.05*
SAP, mmHg	120 ±	2.7	111 ±	2.2^a^		115 ±	1.6^a^	107 ±	1.6^b^		108 ±	2.9^a^	100 ±	2.5^c d e^		F_(1,90)_:18.9	*<0.001*		F_(2,90)_:11.4	*<0.001*		F_(2,90)_:0.06	*>0.05*
DAP, mmHg	76 ±	2.6	67 ±	1.9^a^		69 ±	1.5^a^	65 ±	2.5		65 ±	2.9^a^	58 ±	2.1^c d e^		F_(1,90)_:13.3	*<0.001*		F_(2,90)_:7.78	*<0.001*		F_(2,90)_: 0.69	*>0.05*
MAP, mmHg	93 ±	2.5	84 ±	1.9^a^		87 ±	1.3^a^	82 ±	2.0^b^		83 ±	2.8^a^	75 ±	1.9^c d e^		F_(1,90)_:18.2	*<0.001*		F_(2,90)_:10.8	*<0.001*		F_(2,90)_:0.4	*>0.05*

Values are the means ± S.E.M. BMI, body mass index; SAP, systolic arterial pressure; DAP, diastolic arterial pressure; MAP, mean arterial pressure. ^a^P<0.05 *vs.* Men Low Aerobic Capacity Group; ^b^P<0.05 *vs.* Men Moderate Aerobic Capacity Group; ^c^P<0.05 *vs.* Men High Aerobic Capacity Group; ^d^P<0.05 *vs.* Women Low Aerobic Capacity Group; ^e^P<0.05 *vs.* Women Moderate Aerobic Capacity Group.

**Figure 1:**
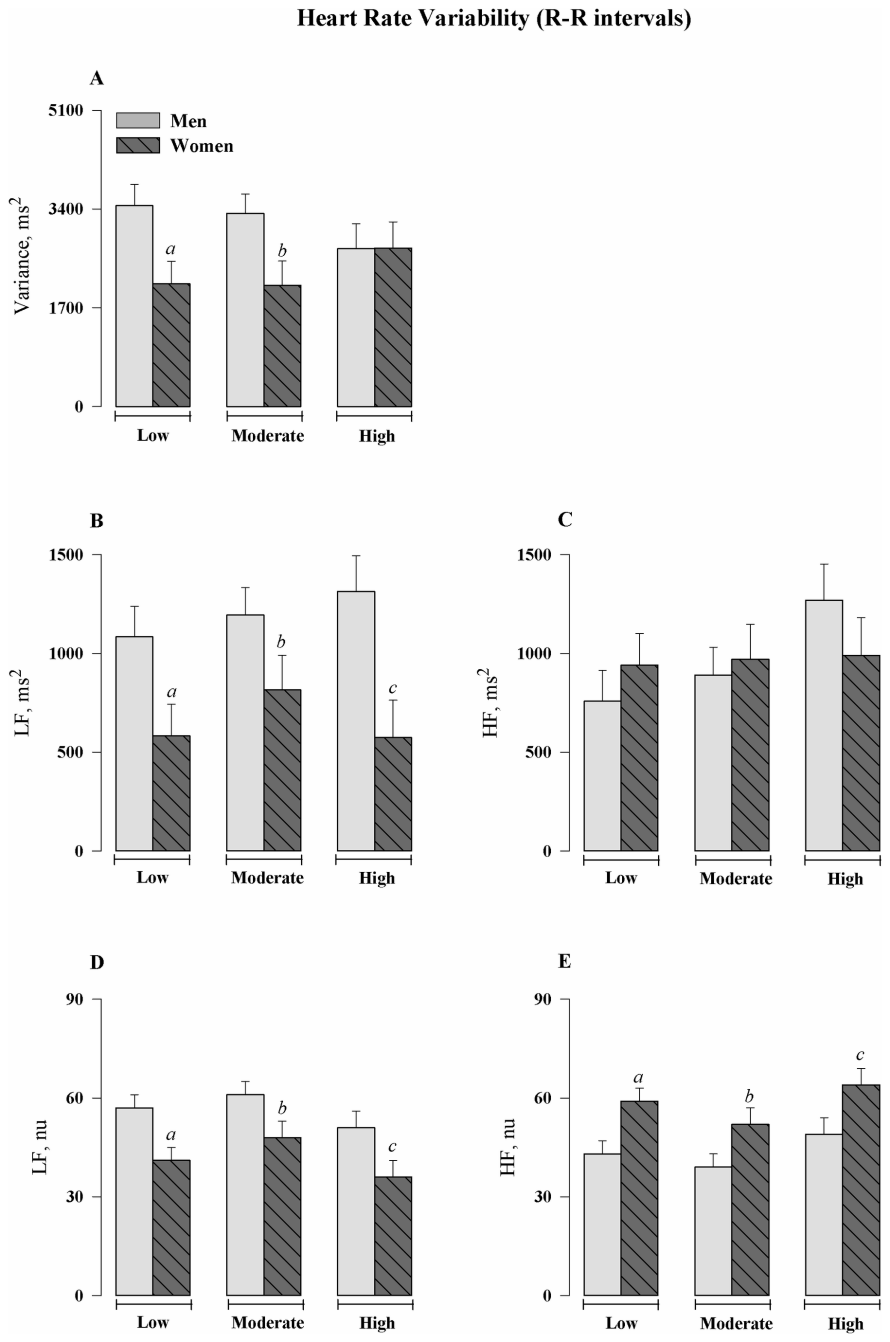
Heart Rate Variability (R-R intervals). (A) Total variance of heart rate obtained by means of series of R–R interval (ms^2^). (B and C) Spectral power density of heart rate in low (LF) and high frequencies (HF) in absolute values. (D and E) Spectral power density of heart rate in the LF and HF bands in normalized units (nu), respectively. Values are means ± S.E.M. ^*a*^P<0.05 *vs*. Men Low Aerobic Capacity Group; ^*b*^P<0.05 *vs*. Men Moderate Aerobic Capacity Group; ^*c*^P<0.05 *vs*. Men High Aerobic Capacity Group.

Table 2 and figure 1 show the results of spectral analysis. Women, regardless of aerobic physical capacity, had a lower total variance (gender factor, F_(1,90)_: 6.89, p=0.010), smaller oscillations in the LF bands in the absolute (gender factor, F_(1,90)_: 15.6, p<0.001) and normalized (gender factor, F_(1,90)_: 15.8, p<0.001) units, and greater oscillations in the HF band in normalized units (gender factor, F_(1,90)_: 15.8, p<0.001) than men. In addition, the groups constituted of women had lower values of LF/HF ratio than men (gender factor, F_(1,90)_: 10.9, p=0.001). In turn, the level of aerobic capacity did not influence any of the spectral parameters evaluated in both men and women.

**Table 2:** Effects of gender and aerobic physical capacity on heart rate variability.

	**Low (N = 35)**					**Moderate (N = 36)**					**High (N = 25)**					**Gender Factor**			**Aerobic Physical Capacity Factor**			**Interaction**	
	**Men (n=18)**		**Women (n=17)**			**Men (n=22)**		**Women (n=14)**			**Men (n=13)**		**Women (n=12)**			**F_(DF)_**	***P***		**F_(DF)_**	***P***		**F_(DF)_**	***P***
**Spectral Parameters**																							
RRi, ms	758 ±	29	755 ±	30		938 ±	26^a^	885 ±	33^d^		1164 ±	34 ^a b^	1119 ±	36 ^d e^		F_(1,90)_:1.7	*>0.05*		F_(2,90)_:70.7	*<0.001*		F_(2,90)_:0.4	*>0.05*
Variance, ms^2^	3456 ±	368	2115 ±	379^a^		3323±	333	2086 ±	418^b^		2716 ±	433	2726 ±	451		F_(1,90)_:6.89	*0.010*		F_(2,90)_:0.02	*>0.05*		F_(2,90)_:1.59	*>0.05*
LF, ms^2^	1084 ±	154	583 ±	159^a^		1194 ±	139	816 ±	175^b^		1314 ±	181	574 ±	189^c^		F_(1,90)_:15.6	*<0.001*		F_(2,90)_:0.60	*>0.05*		F_(2,90)_:0.56	*>0.05*
HF, ms^2^	758 ±	156	940 ±	160		890 ±	141	970 ±	177		1270 ±	183	990 ±	191		F_(1,90)_:0.195	*>0.05*		F_(2,90)_:1.34	*>0.05*		F_(2,90)_:0.93	*>0.05*
LF, nu	57 ±	4	41 ±	4^a^		61 ±	4	48 ±	5^b^		51 ±	5	36 ±	5^c^		F_(1,90)_:15.8	*<0.001*		F_(2,90)_:3.03	*>0.05*		F_(2,90)_:0.07	*>0.05*
HF, nu	43 ±	4	59 ±	4^a^		39 ±	4	52 ±	5^b^		49 ±	5	64 ±	5^c^		F_(1,90)_:15.8	*<0.001*		F_(2,90)_:3.03	*>0.05*		F_(2,90)_:0.07	*>0.05*
LF/HF	1.81 ±	0.31	0.91 ±	0.30^a^		2.09 ±	0.28	1.14 ±	0.35^b^		1.51 ±	0.36	0.69 ±	0.37^c^		F_(1,90)_:10.9	*0.001*		F_(2,90)_:1.17	*>0.05*		F_(2,90)_:0.02	*>0.05*

Values are the means ± S.E.M. LF, low frequency; HF, high frequency; nu, normalized units. ^a^P<0.05 *vs.* Men Low Aerobic Capacity Group; ^b^P<0.05 *vs.* Men Moderate Aerobic Capacity Group; ^c^P<0.05 *vs.* Men High Aerobic Capacity Group; ^d^P<0.05 *vs.* Women Low Aerobic Capacity Group; ^e^P<0.05 *vs.* Women Moderate Aerobic Capacity Group.

## Discussion

This study investigated in detail the HRV and its relationship with aerobic capacity, addressing the differences between genders. The results of the present study show that higher levels of aerobic capacity improve the hemodynamic response, which was demonstrated by lower basal HR, SAP, DAP, and MAP values, independent of gender. However, women presented the lowest pressure values compared to men at each level of aerobic capacity investigated. Regarding the spectral parameters of HRV, the level of aerobic capacity did not induce any difference when same-gender subjects were compared to each other, although sinus bradycardia was observed in the high aerobic capacity group. However, when the comparison was performed between genders, there were differences at each level of physical conditioning.

Recent studies have shown that moderate-to-high cardiorespiratory capacity is related to adaptations of several cardiovascular performance parameters, thus decreasing the risk of cardiac events and mortality [3,23]. In agreement with these studies, the present study shows that aerobic physical training promotes important adaptations, which is addressed below.

### Heart Rate and Arterial Pressure

Aerobic physical training promoted important hemodynamic changes. In the present study, an accentuated sinus bradycardia was observed in high-performance athletes regardless of gender. In fact, sinus bradycardia is a phenomenon observed in athletes as a possible result of autonomic adaptations and/or intrinsic cardiac alterations, mainly involving the sinus node [17,24]. This reduction of heart rate, when associated with athletes, denotes improved performance.

Another favorable effect on cardiovascular health resulting from an increase in aerobic capacity is a decrease in AP. Consistent with the findings reported in the literature, our study shows that the subjects with better aerobic capacity had AP values lower than those with poor aerobic capacity. The mechanisms involved in the physical training effect on AP are not completely elucidated. Some studies suggest the involvement of several mechanisms, such as changes in the activity of the autonomic nervous system, which is mainly characterized by a reduction in the sympathetic influence. An alternative mechanism involves a decrease of serum vasoconstrictor factor levels and an increase in the endothelium-derived dilator factor levels, resulting in a reduction in the peripheral vascular resistance, as evidenced by low levels of norepinephrine and plasmatic renin activity, resulting in an increase in endothelium-induced vasodilatation [25-28].

Regarding the difference in AP response between genders, women showed lower SAP, DAP and MAP values than men, with women in the high aerobic capacity group having AP values smaller than those in other groups. The cause of such a finding is unknown; however, there are studies showing that women exhibit differences in several aspects of hemodynamic regulation compared to men. Among these aspects, one can cite low cardiac responses to baroreceptor activation, low plasmatic renin activity, increased vascular 1-adrenergic response and lower levels of circulating catecholamines compared to men [29-31]. Moreover, several studies indicate that gender differences in the hemodynamic regulation of arterial pressure might be related to sexual hormones [32-35]. In fact, data from the literature suggest that estrogens have a cardioprotective effect in premenopausal women [35-38] because there is a significant increase in the MAP values after menopause, which may be associated with a higher incidence of cardiovascular diseases [34,39,40]. Despite the cardioprotective effect of estrogens, this is not the only factor involved in the AP increase observed after menopause. Another hormone that plays a role in AP regulation is testosterone. Studies report that testosterone, among other factors, induces an increase in MAP by activating the angiotensin-renin system [32-34]. Therefore, testosterone seems to have a harmful effect on MAP, participating more actively in the process of inducing sexual dimorphism.

### Heart Rate Variability

The present study demonstrates that the level of aerobic capacity does not promote changes in the spectral parameters of HRV when same-gender subjects were compared to each other. However, when men were compared to women, differences in each level of aerobic capacity were observed. Women presented the lowest values of LF oscillations and highest values of HF oscillations compared to their male counterparts, regardless of the level of aerobic capacity.

Some studies have demonstrated that analyzing the HRV to quantify the modulating influence of both autonomic components (i.e., sympathetic and parasympathetic) on the heart can also be an important tool in aiding the prognosis of cardiovascular stress and conditioning [41-43]. In fact, this process allows for the non-invasive evaluation of the cardiac autonomic function in different pathophysiological situations, including overtraining syndrome [44,45].

In this sense, the basal indices of cardiac autonomic modulation can be influenced by different conditions, including gender. When genders were compared, regardless of the level of physical conditioning, men presented an autonomic balance in favor of sympathetic modulation characterized by greater LF oscillations, whereas women presented an autonomic balance in favor of HF oscillations. These discrepant results were more evident in well-conditioned subjects (i.e., the moderate and high aerobic capacity groups), showing that gender can indeed influence cardiac autonomic modulation [31,46-48]. In accordance with these observations, the literature has shown that middle-aged women have the greatest variations in the spectral indices of HRV and lowest values of AP at rest conditions compared to men of the same age group. This finding indicates that the female population would present an increased vagal cardiac modulation, which would be evidenced by higher HF values and lower LF values [30,46-48]. However, it was also shown that such a discrepancy in the spectral parameters of HRV decreases between men and women when their age is above 50 years old [46]. It has been suggested that female hormones, especially β-estradiol [49], facilitate vagal cardiac function activation [50]. Therefore, the reduction in the vagal modulation predominantly in women older than 50 years might be attributed to the hypothesis that such a facilitating effect is promoted by female sexual hormones [50]. Conversely, it is speculated that the higher indices of sympathetic autonomic modulation in men might be mainly attributed to their physical constitution, comprised of a greater muscle sympathetic nerve activity [51] as well as a higher number of sympathetic ganglionic neurons compared to women, which would result in a more favorable sympathetic modulation balance [47,48,52].

Regardless of the differences between the genders observed in the present study, several studies have reported that practicing physical exercises regularly improves cardiac autonomic modulation, thus suggesting the hypothesis that athletes or well-conditioned subjects have a better pattern of cardiac autonomic modulation [17,19]. This improvement would be mostly associated with an increase in HRV, which is mainly characterized by a greater influence of the parasympathetic autonomic component on the heart [53-55]. In this way, some studies have shown that athletes possess an HRV greater than that of low-performance subjects [17] and that subjects practicing moderate-to-intense physical activities have an autonomic modulation of HR better than that of sedentary subjects, thus providing evidence of the predominance of vagal modulation (HF) on the sinus node [19]. This finding was also observed when only women were studied [48-55].

However, no alteration in cardiac autonomic modulation was observed in our study when the groups of subjects with different aerobic capacities were compared to each other, regardless of the evaluated gender. Thus, our results suggest that the aerobic capacity level, which is characterized by a greater VO_2peak_ response and low basal HR, did not induce changes in the spectral parameters of HRV, which has also been corroborated by other authors [12,24,56,57]. When this gender comparison was performed, we found no further differences between men and women.

These discrepant results suggest that other components of aerobic physical conditioning may contribute to the difference in cardiac autonomic modulation in healthy subjects. For example, resistance strength is a determinant of aerobic capacity that is independent of the VO_2max_ [58,59]. In addition, although aerobic physical capacity may influence HRV, it does not affect HRV in a dose-dependent manner with increasing levels of physical activity [60].

Meanwhile, other mechanisms may have contributed to these results. Because of the presence of respiratory sinus bradycardia, as evidenced by low values of basal HR in the high aerobic capacity group and the absence of changes in the autonomic modulation of HRV, physical exercise may have played a direct role in the sinus node modulation in these subjects, although this parameter was not investigated in the present study.

## Conclusions

Our results suggest that cardiovascular and autonomic responses, regardless of the level of aerobic capacity, are different between men and women, as the former present a profile of cardiac autonomic modulation that favors the sympathetic component, whereas the latter present a profile that favors the vagal component. In addition, the level of aerobic physical capacity did not interfere with the autonomic modulation of HRV in the healthy subjects, despite the presence of sinus bradycardia in the groups with high aerobic capacity.
